# Supplementary figures and images for: The effects of five weeks of climbing training, on and off the wall, on climbing specific strength, performance, and training experience in female climbers—A randomized controlled trial
Source: PLoS One. 2024 Jul 8;19(7):e0306300. doi: 10.1371/journal.pone.0306300 (PMC11230541; doi:10.1371/journal.pone.0306300)

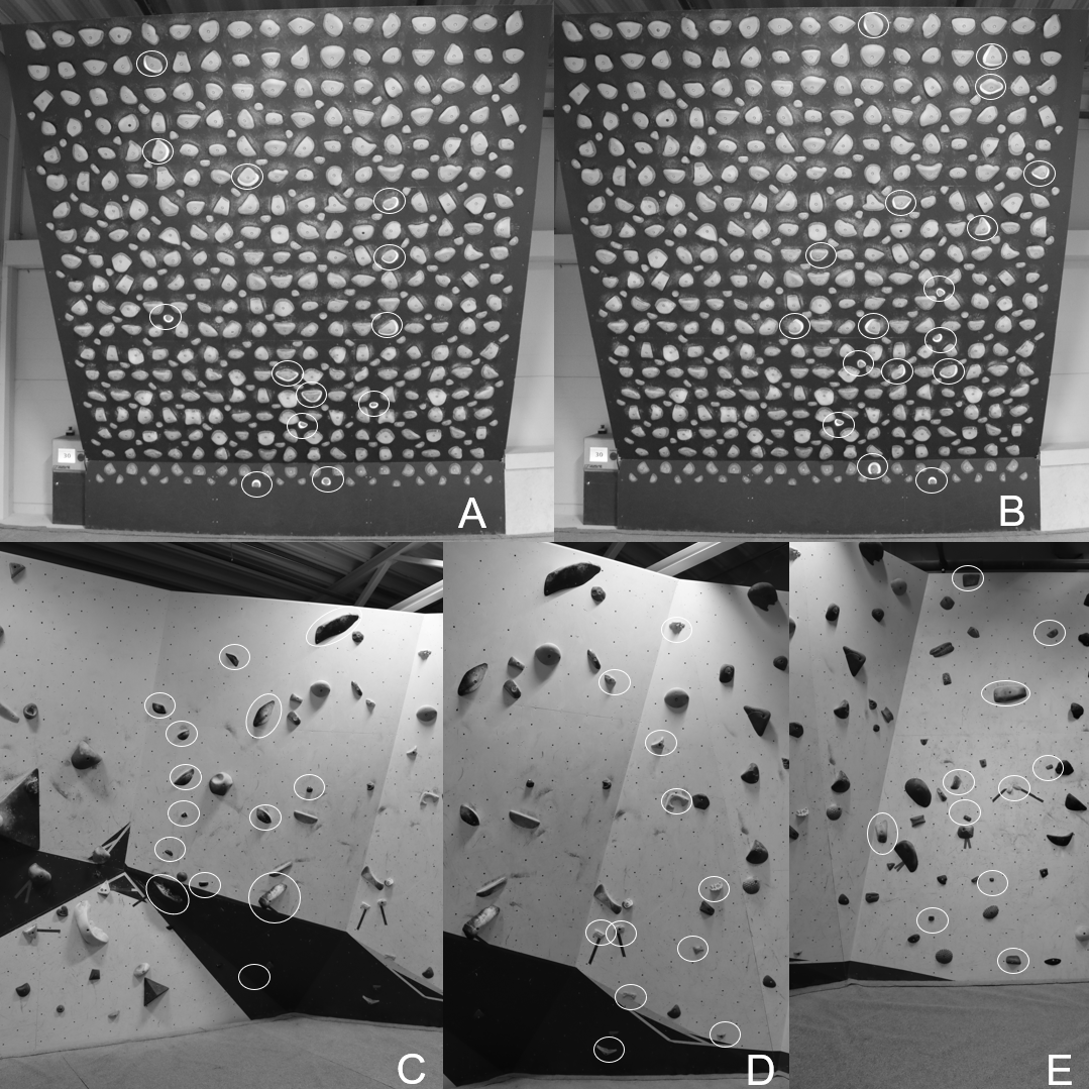

Supplement: S1 Fig — (TIF) [file pone.0306300.s001.tif]

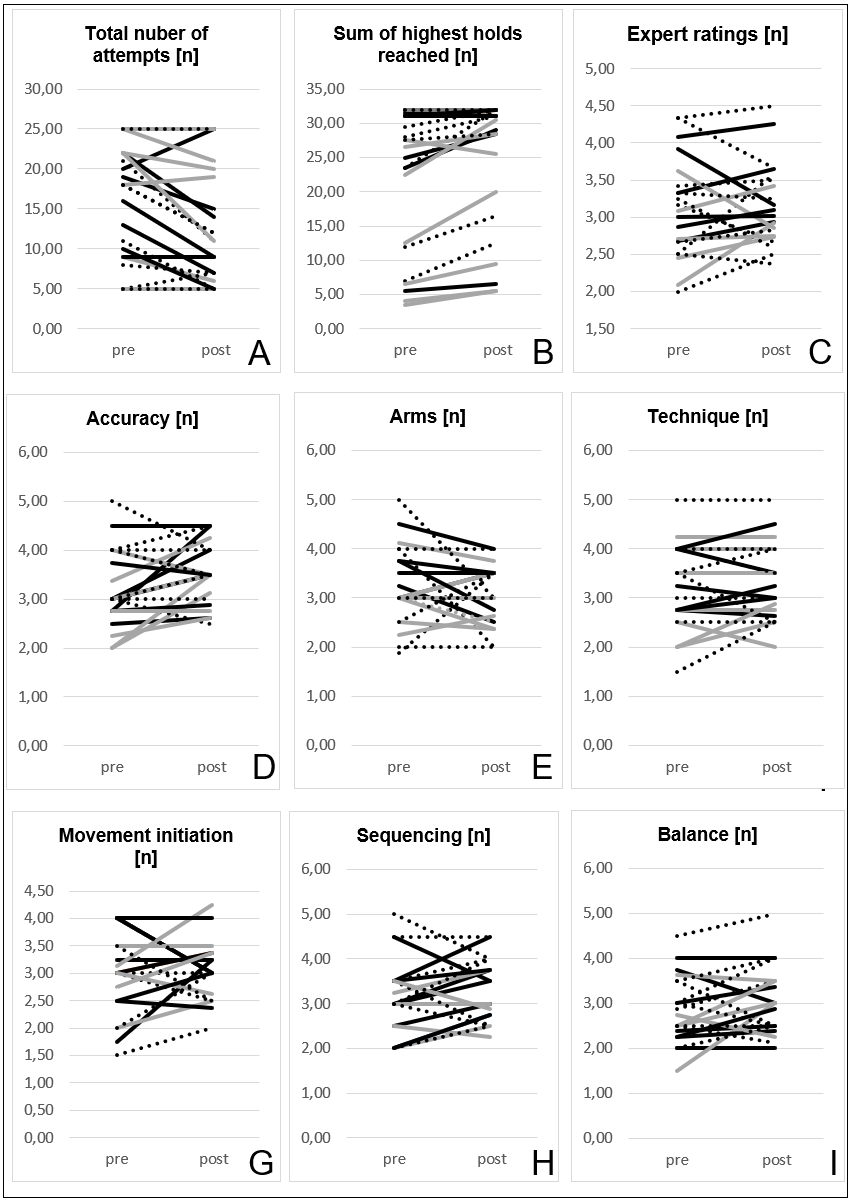

Supplement: S2 Fig — (TIF) [file pone.0306300.s002.tif]

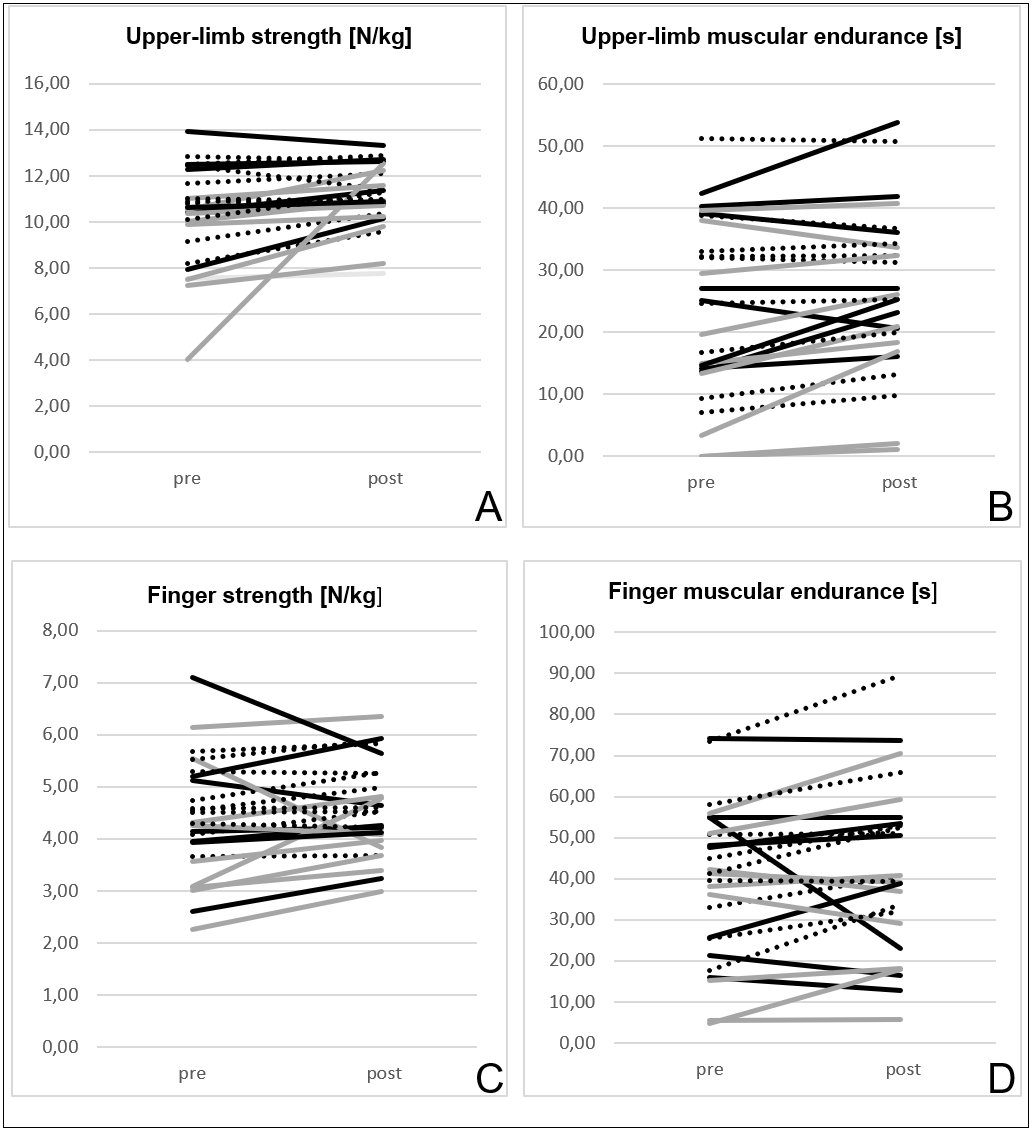

Supplement: S3 Fig — (TIF) [file pone.0306300.s003.tif]

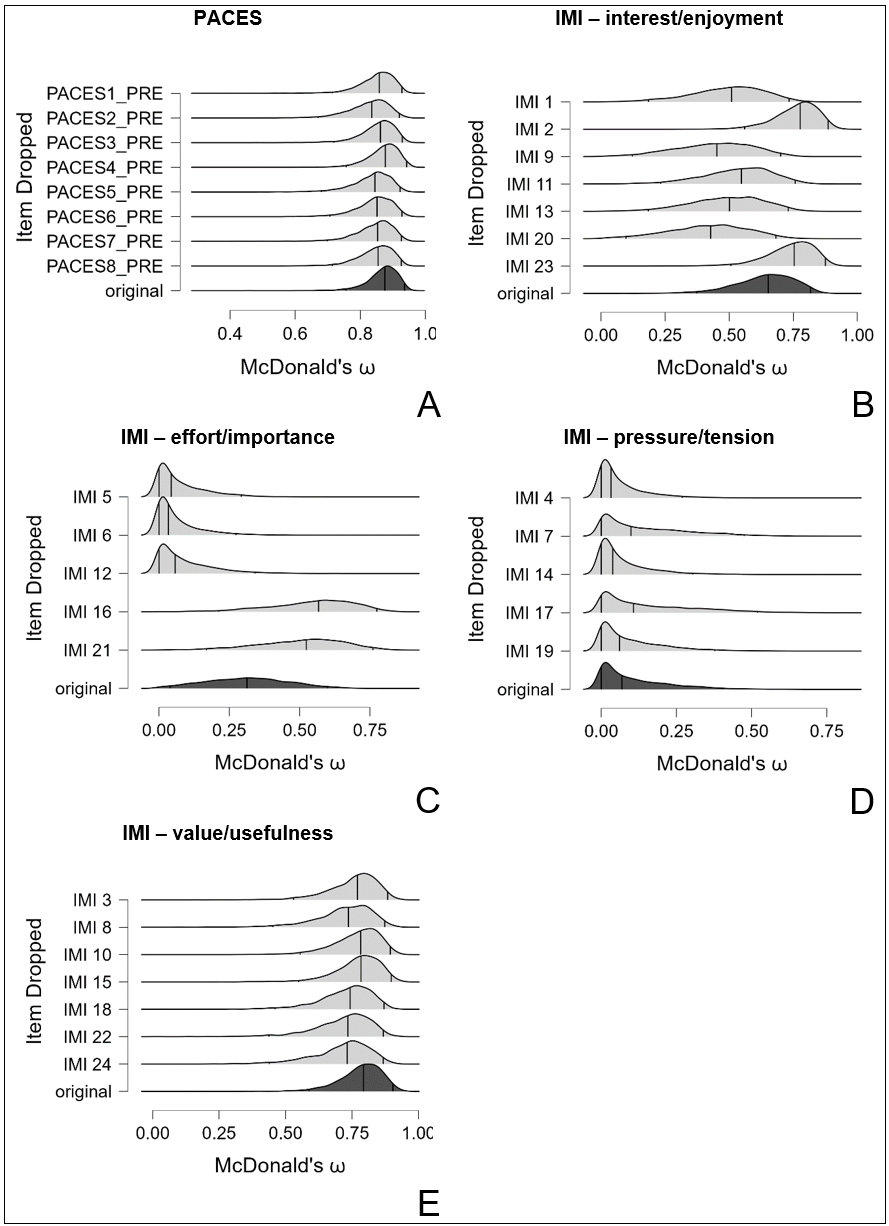

Supplement: S4 Fig — Original values and values in case one item was dropped. PACES–physical activity enjoyment scale, IMI–Intrinsic Motivation Inventory. (TIF) [file pone.0306300.s004.tif]

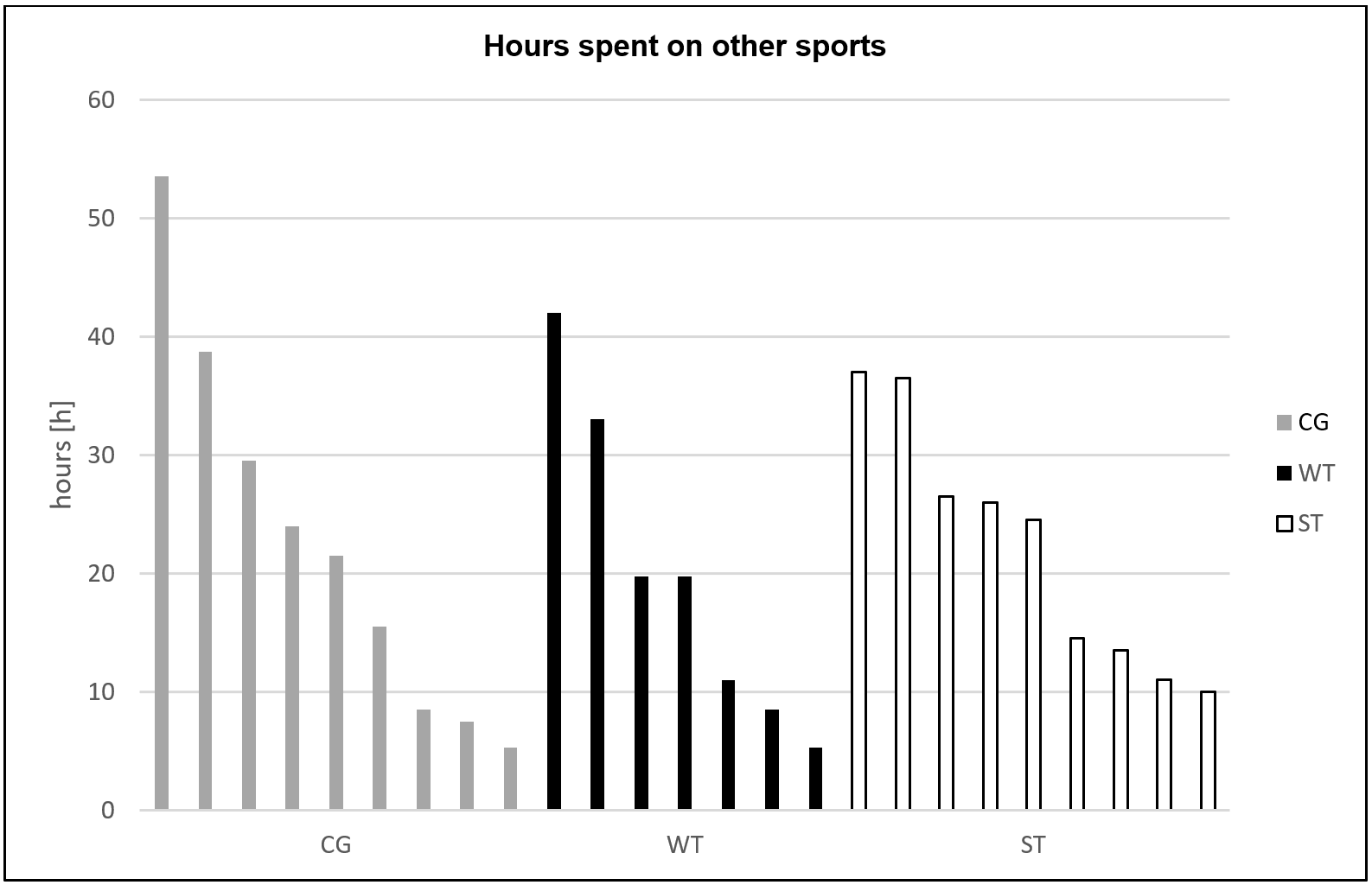

Supplement: S5 Fig — WT–off-the-wall training group, ST–on-the-wall training group, CG–control group. (TIF) [file pone.0306300.s005.tif]
