# Supplementary material for: The effects of five weeks of climbing training, on and off the wall, on climbing specific strength, performance, and training experience in female climbers—A randomized controlled trial
Source: PLoS One. 2024 Jul 8;19(7):e0306300. doi: 10.1371/journal.pone.0306300 (PMC11230541; doi:10.1371/journal.pone.0306300)
Supplement: S1 Table — RPE–rate of perceived exertion, RPD–rate of perceived discomfort, PACES–physical activity enjoyment scale, EES–exercise enjoyment scale, FS–feeling scale, IMI–Intrinsic Motivation Inventory. (PDF) [file pone.0306300.s006.pdf]

S1 Table. Tests and corresponding measures.

Outcome	Test	Measure
Performance	Bouldering – 5 routes:	Sum of highest holds reached (max: 32)
	2 Kilterboard routes (A, B)	Total number of attempts (max: 25)
	3 boulder wall routes (C, D, E)	Expert ratings of best attempt on boulders B and C
Strength	Isometric pull-up	Highest avg. force (N/kg) over 3 s; averaged across three trials
	Bent arm hang	Hangtime [s]
	Finger strength test	Highest avg. force (N/kg) over 3 s; averaged across three trials
	Dead hang	Hangtime [s]
Emotions and motivation	RPE	Total score (max. 10)
	RPD	Total score (max. 10)
	PACES	Total score (max.56)
	EES	Total score (max. 7)
	FS	Total score (max. 5)
	IMI	Average score for each subscale (max. 7)

RPE – rate of perceived exertion, RPD – rate of perceived discomfort, PACES – physical activity enjoyment scale, EES – exercise enjoyment scale, FS – feeling scale, IMI – Intrinsic Motivation Inventory
