# Supplementary material for: The effects of five weeks of climbing training, on and off the wall, on climbing specific strength, performance, and training experience in female climbers—A randomized controlled trial
Source: PLoS One. 2024 Jul 8;19(7):e0306300. doi: 10.1371/journal.pone.0306300 (PMC11230541; doi:10.1371/journal.pone.0306300)
Supplement: S2 Table — (PDF) [file pone.0306300.s007.pdf]

S2 Table. Performance assessment tool.

Video Nr.	Accuracy	Balance/ Fluidity	Sequencing/ Exploration	Technique	Arms	Movement initiation
x<br>⋮						

**Accuracy**	**1** = Messy climbing and must adjust every hand- and foot move.<br>**5** = Hands and feet are placed precisely and accurately every time.
**Balance/ Fluidity**	**1** = Always off balance and moves twitchy and uncontrolled.<br>**5** = Perfect balance and flow in all movements.
**Sequencing/ Exploration**	**1** = Performs sequences inappropriately with a frequent and extended exploration of possible holds.<br>**5** = Does all sequences in an appropriate and effective manner. Finds the right sequence right away with purposeful moves.
**Technique**	**1** = Ineffective and unvaried solutions.<br>**5** = Demonstrates a broad repertoire of skills that are used to solve the boulder appropriately.
**Arms**	**1** = Always climbs with bent arms.<br>**5** = Climbs with straight arms when appropriate.
**Movement initiation**	**1** = All movements are initiated with the arms.<br>**5** = Where possible movements are initiated from lower body or the momentum is maintained.
