# Supplementary material for: The effects of five weeks of climbing training, on and off the wall, on climbing specific strength, performance, and training experience in female climbers—A randomized controlled trial
Source: PLoS One. 2024 Jul 8;19(7):e0306300. doi: 10.1371/journal.pone.0306300 (PMC11230541; doi:10.1371/journal.pone.0306300)
Supplement: S3 Table — Interest/enjoyment–items 1, 2, 9, 11, 13, 20, 23; effort/importance–items 5, 6, 12, 16, 21; pressure/tension–items 4, 7, 14, 17, 19; value usefulness–items 3, 8, 10, 15, 18, 22, 24. (PDF) [file pone.0306300.s008.pdf]

S3 Table. Intrinsic Motivation Inventory.

Items	Lowest score	Highest score
1. I enjoyed doing the strength training very much.	**0**	**7**
2. The strength training did not hold my attention at all.	Not at all true	Very true
3. I would be willing to do this strength training again because it has some value to me.		
4. I felt very tense while doing the strength training.		
5. It was important to me to do well at the strength training.		
6. I tried very hard at the strength training.		
7. I was very relaxed while doing the strength training.		
8. I think that doing this strength training is useful for becoming a better climber.		
9. The strength training was fun to do.		
10. I think this strength training is an important activity.		
11. While I was doing the strength training, I was thinking about how much I enjoyed it.		
12. I put a lot of effort into the strength training.		
13. I would describe the strength training as very interesting.		
14. I felt pressured while doing the strength training.		
15. I think doing this strength training could help me to become a better climber.		
16. I didn't put much energy into the strength training.		
17. I did not feel nervous at all while doing the strength training.		
18. I believe this strength training could be of some value to me.		
19. I was anxious while following the strength training program.		
20. I thought the strength training was quite enjoyable.		
21. I didn't try very hard at the strength training.		
22. I believe doing this strength training could be beneficial to me.		
23. I thought the strength training was a boring activity.		
24. I think the strength training is important to do because it can help me improve my climbing level.		

interest/enjoyment – items 1, 2, 9, 11, 13, 20, 23; effort/importance – items 5, 6, 12, 16, 21; pressure/tension – items 4, 7, 14, 17, 19; value usefulness – items 3, 8, 10, 15, 18, 22, 24
