# Supplementary material for: The effects of five weeks of climbing training, on and off the wall, on climbing specific strength, performance, and training experience in female climbers—A randomized controlled trial
Source: PLoS One. 2024 Jul 8;19(7):e0306300. doi: 10.1371/journal.pone.0306300 (PMC11230541; doi:10.1371/journal.pone.0306300)
Supplement: S4 Table — WT–off-the-wall training group, ST–on-the-wall training group, CG–control group. (PDF) [file pone.0306300.s009.pdf]

S4 Table. Number of completed training sessions and hours spent climbing and bouldering during the five-week intervention by each participant.

Participant number	Group	Training sessions	Climbing [h]
**1**	ST	10	13,00
**3**	WT	12	27,75
**4**	WT	12	13,75
**5**	WT	10	35,25
**6**	WT	10	10,00
**7**	WT	10	23,00
**8**	CG	.	19,50
**9**	WT	10	12,50
**10**	ST	10	20,00
**11**	CG	.	11,50
**12**	ST	11	41,00
**14**	CG	.	32,00
**15**	CG	.	26,75
**16**	CG	.	9,50
**18**	WT	11	25,75
**19**	ST	10	16,50
**20**	ST	10	22,50
**21**	CG	.	2,25
**22**	CG	.	20,50
**25**	CG	.	19,50
**26**	CG	.	17,50
**27**	ST	10	12,00
**28**	ST	14	52,00
**29**	WT	10	20,75
**30**	ST	10	30,50
**31**	ST	10	9,50

WT – off-the-wall training group, ST – on-the-wall training group, CG – control group
