# Supplementary material for: The effects of five weeks of climbing training, on and off the wall, on climbing specific strength, performance, and training experience in female climbers—A randomized controlled trial
Source: PLoS One. 2024 Jul 8;19(7):e0306300. doi: 10.1371/journal.pone.0306300 (PMC11230541; doi:10.1371/journal.pone.0306300)
Supplement: S5 Table — (PDF) [file pone.0306300.s010.pdf]

S5 Table. Training diary (CG did not fill in the last column).

Date	Type of training (climbing, cross fit, soccer, running, biking, hiking, etc.)	How long?	How hard (0-10)? 0 = not hard at all 10 = extremely exhausting	Intensity of prescribed training and percentage of prescribed training (if not completed)
…	…	…	…	…
