# Supplementary material for: The effects of five weeks of climbing training, on and off the wall, on climbing specific strength, performance, and training experience in female climbers—A randomized controlled trial
Source: PLoS One. 2024 Jul 8;19(7):e0306300. doi: 10.1371/journal.pone.0306300 (PMC11230541; doi:10.1371/journal.pone.0306300)
Supplement: S6 Table — CG–control group, WT–on -the-wall training group, ST–off-the -wall training group. (PDF) [file pone.0306300.s011.pdf]

S6 Table. Pre- and post-test results for all strength and performance tests.

Test	pre as [N/kg]			pre bent arm hang [s]			pre fs [N/kg]			pre dead hang [s]		
**Group**	**CG**	**ST**	**WT**	**CG**	**ST**	**WT**	**CG**	**ST**	**WT**	**CG**	**ST**	**WT**
**Mean**	8.694	10.986	11.145	17.591	27.204	27.034	3.918	4.687	4,599	32.279	42.680	42.852
**Std. Deviation**	2.280	1.588	1.776	15.394	14.304	12.311	1.275	0.689	1.307	19.053	16.919	19.943

Test	post as [N/kg]			post bent arm hang [s]			post fs [N/kg]			post dead hang [s]		
**Group**	**CG**	**ST**	**WT**	**CG**	**ST**	**WT**	**CG**	**ST**	**WT**	**CG**	**ST**	**WT**
**Mean**	10.459	11.366	11.615	21.302	28.154	30.480	4.213	4.836	4.669	35.337	50.863	40.454
**Std. Deviation**	1.654	1.064	1.131	13.639	12.675	12.554	0.994	0.729	0.890	20.538	18.059	21.415

Test	pre number of attempts [n]			pre best hold [n]			pre expert ratings [n]		
**Group**	**CG**	**ST**	**WT**	**CG**	**ST**	**WT**	**CG**	**ST**	**WT**
**Mean**	19.556	18.556	24.833	26.313	15.111	14.250	2.743	3.313	3.200
**Std. Deviation**	7.552	11.941	9.203	9.008	8.054	5.994	0.544	0.778	0.606

Test	post number of attempts [n]			post best hold [n]			post expert ratings [n]		
**Group**	**CG**	**ST**	**WT**	**CG**	**ST**	**WT**	**CG**	**ST**	**WT**
**Mean**	17.444	12.111	11.125	21.000	27.611	27.750	2.842	3.188	3.387
**Std. Deviation**	8.064	7.801	6.728	11.305	7.586	8.693	0.339	0.686	0.468

CG – control group, WT – on -the-wall training group, ST – off-the -wall training group
