# Supplementary material for: The effects of five weeks of climbing training, on and off the wall, on climbing specific strength, performance, and training experience in female climbers—A randomized controlled trial
Source: PLoS One. 2024 Jul 8;19(7):e0306300. doi: 10.1371/journal.pone.0306300 (PMC11230541; doi:10.1371/journal.pone.0306300)
Supplement: S7 Table — BF10 –Bayes factor (evidence for the alternative hypothesis relative to the null hypothesis/null model). (PDF) [file pone.0306300.s012.pdf]

S7 Table. Inter-rater reliability.

		Rater 1	Rater 2	Rater 3	Rater 4	Rater 5
**Rater 1**	Pearson's r	–				
	BF_{10}	–				
**Rater 2**	Pearson's r	0.741	–			
	BF_{10}	5.402	–			
**Rater 3**	Pearson's r	0.493	0.453	–		
	BF_{10}	0.984	0.836	–		
**Rater 4**	Pearson's r	0.585	0.740	0.638	–	
	BF_{10}	1.561	5.334	2.203	–	
**Rater 5**	Pearson's r	0.694	0.531	0.560	0.727	–
	BF_{10}	3.421	1.169	1.358	4.651	–

BF_{10} – Bayes factor (evidence for the alternative hypothesis relative to the null hypothesis/null model)
