# Supplementary material for: The effects of five weeks of climbing training, on and off the wall, on climbing specific strength, performance, and training experience in female climbers—A randomized controlled trial
Source: PLoS One. 2024 Jul 8;19(7):e0306300. doi: 10.1371/journal.pone.0306300 (PMC11230541; doi:10.1371/journal.pone.0306300)
Supplement: S8 Table — BF10 –Bayes factor (evidence for the alternative hypothesis relative to the null hypothesis/null model). (PDF) [file pone.0306300.s013.pdf]

S8 Table. Intra-rater reliability.

Rater	Pearson's r	BF_{10}
Rater 1	.185	0.360
Rater 2	.711	316.709
Rater 3	.806	10514.590
Rater 4	.655	$1.062*10^{6}$
Rater 5	.857	$4.571*10^{13}$

BF_{10} – Bayes factor (evidence for the alternative hypothesis relative to the null hypothesis/null model)
