# Supplementary material for: The effects of five weeks of climbing training, on and off the wall, on climbing specific strength, performance, and training experience in female climbers—A randomized controlled trial
Source: PLoS One. 2024 Jul 8;19(7):e0306300. doi: 10.1371/journal.pone.0306300 (PMC11230541; doi:10.1371/journal.pone.0306300)
Supplement: S9 Table — A decrease in the total number of attempts was rated as a performance increase.––performance decrease, ○ –no performance increase, +–performance increase, WT–off-the-wall training group, ST–on-the-wall training group, CG–control group. (PDF) [file pone.0306300.s014.pdf]

S9 Table. Individual development from pre- to post-test across groups and tests. A decrease in the total number of attempts was rated as a performance increase.

Variable	Group	–	○	+
**Upper-limb strength**	WT	1	-	6
	ST	3	-	6
	CG	-	-	9
**Finger strength**	WT	2	-	6
	ST	2	1	6
	CG	2	-	7
**Upper-limb local muscular endurance**	WT	4	1	3
	ST	3	-	6
	CG	1	-	8
**Figer local muscular endurance**	WT	4	1	3
	ST	1	-	8
	CG	3	-	6
**Sum of highest holds reached**	WT	1	2	5
	ST	-	3	6
	CG	1	2	6
**Total number of attempts**	WT	1	2	5
	ST	1	3	4
	CG	1	4	4
**Expert ratings**	WT	1	-	6
	ST	3	-	5
	CG	1	-	4

– – performance decrease, ○ – no performance increase , **+** – performance increase, WT – off-the-wall training group, ST – on-the-wall training group, CG – control group
