# Supplementary material for: The effects of five weeks of climbing training, on and off the wall, on climbing specific strength, performance, and training experience in female climbers—A randomized controlled trial
Source: PLoS One. 2024 Jul 8;19(7):e0306300. doi: 10.1371/journal.pone.0306300 (PMC11230541; doi:10.1371/journal.pone.0306300)
Supplement: S10 Table — RPE–Rate of perceived exertion, RPD–rate of perceived discomfort, FS–feeling scale, EES–exercise enjoyment scale, PACES–physical activity enjoyment scale, IMI–Intrinsic Motivation Inventory, CG–control group, WT–on-the-wall training group, ST–off-the-wall training group, SD–standard deviation. (PDF) [file pone.0306300.s015.pdf]

S10 Table. Results for all emotional and motivational measurements.

Measurement	Time	Group	Mean	SD
RPE	Pre	WT	5.750	1.282
		ST	5.111	1.364
	Mid	WT	6.750	1.035
		ST	6.778	1.394
	Post	WT	6.750	1.035
		ST	6.889	1.364
RPD	Pre	WT	2.875	1.885
		ST	3.222	1.716
	Mid	WT	4.750	1.669
		ST	4.556	1.667
	Post	WT	4.000	1.927
		ST	5.111	2.028
FS	Pre	WT	3.500	0.756
		ST	2.111	1.900
	Mid	WT	3.750	1.035
		ST	2.333	1.936
	Post	WT	3.875	0.991
		ST	2.444	1.424
EES	Pre	WT	6.000	0.756
		ST	5.111	0.601
	Mid	WT	5.750	1.389
		ST	4.889	0.928
	Post	WT	5.500	0.926
		ST	4.333	0.866
PACES	Pre	WT	51.125	4.970
		ST	40.778	5.380
	Mid	WT	48.250	5.994
		ST	38.889	5.419
	Post	WT	48.375	5.680
		ST	37.444	4.003
IMI – Interest/ Enjoyment	Post	WT	6.125	0.746
		ST	4.871	1.023
IMI – Effort/ Importance	Post	WT	6.475	0.453
		ST	5.222	1.222
IMI – Value/ Usefulness	Post	WT	6.466	0.606
		ST	5.650	0.867
IMI – Pressure/ Tension	Post	WT	3.200	0.938
		ST	2.822	0.961

RPE – Rate of perceived exertion, RPD – rate of perceived discomfort, FS – feeling scale, EES – exercise enjoyment scale, PACES – physical activity enjoyment scale, IMI – Intrinsic Motivation Inventory, CG – control group, WT – on-the-wall training group, ST – off-the-wall training group, SD – standard deviation
