# Supplementary material for: The effects of five weeks of climbing training, on and off the wall, on climbing specific strength, performance, and training experience in female climbers—A randomized controlled trial
Source: PLoS One. 2024 Jul 8;19(7):e0306300. doi: 10.1371/journal.pone.0306300 (PMC11230541; doi:10.1371/journal.pone.0306300)
Supplement: S11 Table — RPD–rate of perceived discomfort, EES–exercise enjoyment scale, FS–feeling scale, PACES–physical activity enjoyment scale, BF10 –Bayes factor (evidence for the alternative hypothesis relative to the null hypothesis. (PDF) [file pone.0306300.s016.pdf]

S11 Table. Correlation between items of different scales.

Items	Pearson's r	BF_{10}
PACES 1 - EES	.852	1677.231
PACES 1 – IMI 1	.713	34.442
EES – IMI 1	.720	39.447
PACES 2 – IMI 23	-.619	7.684
PACES 4 - RPD	.163	0.359
PACES 5 – IMI 9	.579	4.656
PACES 6 - FS	.705	29.463

RPD – rate of perceived discomfort, EES – exercise enjoyment scale, FS – feeling scale, PACES – physical activity enjoyment scale, BF_{10} – Bayes factor (evidence for the alternative hypothesis relative to the null hypothesis
