# Supplementary material for: The effects of five weeks of climbing training, on and off the wall, on climbing specific strength, performance, and training experience in female climbers—A randomized controlled trial
Source: PLoS One. 2024 Jul 8;19(7):e0306300. doi: 10.1371/journal.pone.0306300 (PMC11230541; doi:10.1371/journal.pone.0306300)
Supplement: S12 Table — (PDF) [file pone.0306300.s017.pdf]

Nr.	Group	Age	Height [cm]	Weight [kg]	Climbing experience [yrs]	Training/wk [h]	best RP	best OS	pre as [N]	pre bent arm hang [s]
**1**	ST	19	170	64,5	1	2,0	13,5	13,5	530,50	7,09
**3**	WT	21	166	61,3	2	3,5	18	16	639,30	14,11
**4**	WT	25	188	89,1	1	2,0	15	14	708,80	13,63
**5**	WT	22	166	47,7	1	3,0	16	15	516,15	39,15
**6**	WT	23	165	64,1	3	3,0	15	15	800,46	40,31
**7**	WT	23	163	74,00	3,5	2,5	18	14	786,82	25,08
**8**	CG	23	169	67,8	5	3,0	17	16	681,12	14,89
**9**	WT	31	164	56,8	8	2,5	18	16	696,62	27,10
**10**	ST	28	165	55,5	5	1,0	16	13,5	507,96	9,21
**11**	CG	26	166	62,2	2	2,0	16	13,5	644,79	13,35
**12**	ST	25	168	61,2	4	4,0	18	16	714,37	32,02
**14**	CG	23	170	66,5	2	3,0	17	16	267,30	38,07
**15**	CG	21	169	68,0	0,3	4,0	13,5	13,5	513,87	0,01
**16**	CG	20	166	72,7	2	2,5	15	13,5	765,60	39,64
**18**	WT	23	175	71,4	1,5	2,0	16	13,5	759,98	14,60
**19**	ST	21	170	59,9	4	2,5	16	13,5	768,97	51,18
**20**	ST	28	171	67,0	3	3,0	15	15	835,11	32,22
**21**	CG	32	171	68,0	1	0,5	13,5	13,5	674,17	19,66
**22**	CG	21	166	76,0	1	1,5	13,5	13,5	571,09	3,32
**25**	CG	25	167	62,3	0,3	2,5	13,5	12	452,26	0,01
**26**	CG	22	165	59,8	0,3	4,0	16	13,5	659,97	29,37
**27**	ST	25	167	68,3	1	1,5	16	16	743,41	24,65
**28**	ST	25	170	62,9	4,5	3,5	18	16	786,72	38,80
**29**	WT	25	164	51,3	1	2,0	15	14	714,81	42,29
**30**	ST	22	174	64,9	1	2,5	18	16	715,23	32,99
**31**	ST	25	174	60,3	3	1,5	14	12	610,29	16,68

pre fs [N]	pre dead hang [s]	post as [N]	post bent arm hang [s]	post fs [N]	post dead hang [s]	pre number of tries	post number of tries	pre best hold	post best hold
235,80	32,92	619,66	9,70	238,04	40,57	25,00	25,00	7,00	12,50
290,38	47,71	698,09	16,03	323,78	53,36	13,00	7,00	31,00	32,00
231,78	16,02	903,23	23,10	289,28	12,90	20,00	25,00	5,50	6,50
197,85	74,04	524,68	36,11	201,56	73,65	5,00	5,00	32,00	32,00
327,75	54,95	810,14	41,86	297,60	23,05	22,00	14,00	23,50	29,00
292,33	21,43	800,02	20,65	314,89	16,58	19,00	15,00	25,00	28,50
375,38	41,42	741,19	18,30	259,52	39,20	9,00	6,00	32,00	32,00
295,57	54,84	722,03	27,10	336,23	54,84	9,00	9,00	31,00	31,00
226,45	17,68	575,73	13,21	251,68	33,83	21,00	11,00	23,50	32,00
265,01	38,20	668,07	20,87	253,89	40,82	18,00	19,00	27,50	25,50
324,44	50,72	742,01	31,25	321,75	51,01	5,00	7,00	32,00	32,00
408,81	55,95	835,04	33,59	422,54	70,50	5,00	5,00	32,00	32,00
210,03	5,50	528,73	2,00	325,29	5,77	25,00	25,00	4,00	5,50
219,34	42,25	889,52	40,74	268,33	37,03	25,00	21,00	12,50	20,00
281,00	25,77	778,54	25,21	294,90	38,80	16,00	9,00	31,50	31,00
331,23	41,19	758,51	50,73	352,77	52,51	8,00	7,00	32,00	32,00
304,11	45,06	765,45	32,34	333,71	53,08	18,00	12,00	28,00	31,00
242,06	36,15	698,06	26,00	270,47	29,18	22,00	20,00	26,50	28,50
232,72	15,22	746,09	16,82	257,88	18,17	25,00	25,00	6,50	9,50
141,02	4,84	510,88	1,00	186,77	17,93	25,00	25,00	3,50	5,50
258,45	50,98	692,85	32,40	288,15	59,43	22,00	11,00	22,50	30,50
308,07	39,58	749,85	25,23	309,35	39,40	18,00	12,00	27,50	28,50
356,80	73,53	812,20	36,67	366,70	89,43	5,00	5,00	32,00	32,00
364,11	48,06	683,57	53,78	289,39	50,45	10,00	5,00	31,00	32,00
278,36	58,06	708,44	34,25	273,47	65,99	11,00	5,00	29,50	32,00
277,33	25,38	681,48	20,01	277,50	31,95	25,00	25,00	12,00	16,50

pre accuracy	post accuracy	pre balance	post balance	pre sequencing	post sequencing	pre technique	post technique	pre arms
3,00	3,00	2,00	2,00	3,00	3,00	3,00	3,00	4,00
2,50	2,60	2,50	2,50	2,50	3,00	2,70	2,70	3,10
.	.	.	.	.	.	.	.	.
3,00	4,00	3,00	3,30	2,83	3,70	3,67	3,90	3,67
2,70	4,17	2,80	3,33	2,80	3,33	2,60	3,17	1,90
3,50	3,33	3,50	2,83	4,17	3,17	3,67	3,33	2,83
3,30	4,00	3,60	3,33	3,20	3,33	4,20	4,00	4,10
4,00	4,33	4,00	4,33	3,00	4,33	3,33	4,33	4,33
.	.	.	.	.	.	.	.	.
3,00	3,50	2,50	3,50	3,00	3,00	3,50	3,50	3,00
4,50	4,00	3,50	4,00	4,50	4,00	4,00	4,00	4,00
.	.	.	.	.	.	.	.	.
2,00	3,50	1,50	3,00	2,00	2,50	2,00	2,50	3,00
2,00	3,20	2,17	3,10	2,00	2,70	2,67	2,60	3,00
5,00	2,80	4,00	2,30	6,00	3,60	5,00	2,90	7,00
4,00	3,50	3,50	4,00	3,50	4,00	3,50	4,00	3,00
3,00	2,50	3,50	2,50	3,50	2,50	3,50	2,50	3,00
2,67	2,70	2,50	2,20	3,33	2,80	2,00	2,80	2,50
.	.	.	.	.	.	.	.	.
.	.	.	.	.	.	.	.	.
2,17	2,60	2,50	2,10	2,50	2,10	2,33	1,90	2,33
3,00	3,00	3,00	2,50	3,00	2,50	2,50	2,50	2,50
4,00	4,50	4,50	5,00	4,50	4,50	5,00	5,00	4,00
3,50	3,50	2,00	2,90	2,00	2,70	3,17	3,00	3,33
4,00	4,00	2,50	2,50	3,00	4,00	4,00	3,50	3,00
3,00	3,50	2,00	2,50	2,00	2,50	1,50	2,50	2,00

post arms	pre movement initiation	post movement initiation	climbing [h]	pre RPE	mid RPE	post RPE	pre RPD	mid RPD	post RPD	pre FS	mid FS	post FS	pre EES	mid EES
2,00	4,00	3,00	13,00	6	8	7	3	7	7	3	-1	-1	4	5
3,40	3,00	3,30	27,75	6	6	7	1	4	6	3	4	3	5	7
.	.	.	13,75	8	7	7	5	8	5	3	5	3	6	5
3,50	3,33	3,30	35,25	5	7	6	5	5	5	3	4	3	6	7
3,33	1,80	3,17	5,00	7	5	8	4	3	1	4	4	5	6	6
2,33	3,50	3,00	23,00	5	8	6	2	5	1	4	2	5	7	3
3,50	3,20	4,00	19,50	.	.	.	.	.	.	.	.	.	.	.
4,33	4,00	4,33	12,50	4	7	8	0	4	5	3	3	3	5	5
.	.	.	20,00	5	7	7	3	4	6	3	3	4	5	6
3,50	3,50	3,50	11,50	.	.	.	.	.	.	.	.	.	.	.
2,50	3,50	3,00	41,00	4	7	7	2	3	6	0	3	3	5	5
.	.	.	32,00	.	.	.	.	.	.	.	.	.	.	.
3,50	2,00	2,50	26,75	.	.	.	.	.	.	.	.	.	.	.
2,30	2,50	2,50	9,50	.	.	.	.	.	.	.	.	.	.	.
2,60	5,50	3,20	25,75	6	8	7	2	6	5	5	5	5	7	7
2,50	3,00	3,00	16,50	5	7	7	5	6	6	3	0	3	5	3
3,00	3,00	2,50	22,50	5	4	4	5	3	3	-1	1	3	6	5
2,30	2,83	3,30	2,25	.	.	.	.	.	.	.	.	.	.	.
.	.	.	20,50	.	.	.	.	.	.	.	.	.	.	.
.	.	.	19,50	.	.	.	.	.	.	.	.	.	.	.
2,60	2,83	2,50	17,50	.	.	.	.	.	.	.	.	.	.	.
3,50	2,00	3,00	12,00	7	7	6	5	5	3	4	3	2	5	4
4,00	4,00	4,00	52,00	4	5	7	4	5	5	3	3	3	6	6
3,50	2,50	3,00	20,75	5	6	5	4	3	4	3	3	4	6	6
3,00	3,50	2,50	30,50	3	8	9	2	6	8	0	5	3	5	5
2,00	1,50	2,00	9,50	7	8	8	0	2	2	4	4	2	5	5

post EES	pre PACES	mid PACES	post PACES	Interest/ Enjoyment	Efort/ Importance	Pressure/ Tension	Value/ Usefulness
4	35	35	35	6,86	6,60	1,20	6,86
6	53	53	54	6,86	6,60	1,20	6,86
5	45	39	42	5,43	5,60	3,60	6,86
6	54	55	53	6,43	6,80	4,60	6,29
6	51	51	53	6,71	6,20	3,20	7,00
5	54	44	44	5,86	6,60	3,40	6,43
.	.	.	.	.	.	.	.
4	56	48	46	6,29	7,00	3,20	6,86
6	40	42	40	5,00	5,40	3,80	5,86
.	.	.	.	.	.	.	.
5	43	49	44	5,57	4,00	4,00	5,71
.	.	.	.	.	.	.	.
.	.	.	.	.	.	.	.
.	.	.	.	.	.	.	.
7	54	54	54	6,71	6,80	3,20	6,29
4	50	36	42	4,14	6,60	2,80	6,14
4	36	34	34	4,00	4,20	2,40	4,57
.	.	.	.	.	.	.	.
.	.	.	.	.	.	.	.
.	.	.	.	.	.	.	.
.	.	.	.	.	.	.	.
3	39	38	33	4,14	5,40	2,80	5,57
5	45	40	38	5,71	6,80	2,00	6,14
5	42	42	41	4,71	6,20	3,20	5,14
4	34	32	38	3,71	3,80	4,00	6,00
4	45	44	33	4,71	4,20	2,40	4,00
