# Supplementary material for: The effects of five weeks of climbing training, on and off the wall, on climbing specific strength, performance, and training experience in female climbers—A randomized controlled trial
Source: PLoS One. 2024 Jul 8;19(7):e0306300. doi: 10.1371/journal.pone.0306300 (PMC11230541; doi:10.1371/journal.pone.0306300)
Supplement: Attachment — Submitted filename: Response to Reviewers.docx [file pone.0306300.s018.docx]

**Response to Reviewers**

Reviewer #1:

Thank you for your comments! Especially the first has helped to increase the quality of our paper.

- Please mark significances in diagrams. – Significance was included in figures 7 and 8. As MANCOVAs were calculated for strength and performance tests, no differences were determined for the groups regarding the individual tests. Significance can therefore not be added to figures 5 and 6.
- Please omit lines of tables and mark alternative rows by colors – This was implemented as proposed.

Reviewer #2:

Thank you for your thorough review! In particular, the readability of our paper was greatly improved through your feedback.

- The type of study should be written in the title – The title was changed accordingly.
- The introduction should be shortened. The article as a whole is very long and this amount of expansion and dispersion hinders the transmission of the main goal and the valuable findings of the research. – The introduction was shortened.
- How did you calculate the sample size (31 cases)? – We added sufficient sample size as proposed by Brysbaert to the study design section. The town where the study was conducted is very small and has a very small climbing community. Therefore, we were not able to meet the criteria, but tried to recruit as many subjects as possible. Study power is discussed in the discussion.
- Randomization is not adequately explained. – The randomization process is described in detail in the methods section. We have highlighted the respective section with a comment in the tracked manuscript.
- RCTs should be registered and the registration number should be inserted. – You are right, the study should have been registered in advance to ensure additional quality. This was neglected by the authors. Nevertheless, the study was thoroughly evaluated by an ethics commission. Unfortunately, registering a study after it has been completed is not in line with ICMJE recommendations, which is why we have decided against registering the study retrospectively. However, we will take the comment into account and consider preregistration in future studies.
- Abbreviations should be written below each table. – This was implemented as proposed.
- There are too many tables. Most of the tables should be moved to the supplementary file (especially tables 2-7). – Some of the tables suggested and a few others (5 in total) were moved to the supplementary file. The total number of tables was reduced to 11
- Do the graphs replicate the data in the tables? If the answer is yes, what was the need to repeat? It is better not to repeat data in results. – Tables repeating the data were moved to the supplementary material. It was important for us to provide the exact data but we see the point in not repeating the results in the paper itself.
- In general, the article is very long, scattered, and of course valuable data, and specialized in sports, which seems to be more suitable for specialized sports journals. – Climbing research is still very young. PLOS ONE, in particular, has published many important findings in the field of climbing and thus supports a basis for research in this area. We have decided to submit our paper here in order to reach the climbing community in research and practice in the best possible way, and would like to stick to this submission for the reasons mentioned above.
